# Phenomic data-facilitated rust and senescence prediction in maize using machine learning algorithms

**DOI:** 10.1038/s41598-022-11591-0

**Published:** 2022-05-09

**Authors:** Aaron J. DeSalvio, Alper Adak, Seth C. Murray, Scott C. Wilde, Thomas Isakeit

**Affiliations:** 1 Department of Biochemistry & Biophysics, Texas A&M University, College Station, TX 77843-2128 USA; 2 Department of Soil and Crop Sciences, Texas A&M University, College Station, TX 77843-2474 USA; 3 Department of Plant Pathology and Microbiology, Texas A&M University, College Station, TX 77843-2474 USA

**Keywords:** Plant breeding, Plant development, Plant genetics, Plant physiology, Plant stress responses

## Abstract

Current methods in measuring maize (*Zea mays* L.) southern rust (*Puccinia polyspora* Underw.) and subsequent crop senescence require expert observation and are resource-intensive and prone to subjectivity. In this study, unoccupied aerial system (UAS) field-based high-throughput phenotyping (HTP) was employed to collect high-resolution aerial imagery of elite maize hybrids planted in the 2020 and 2021 growing seasons, with 13 UAS flights obtained from 2020 and 17 from 2021. In total, 36 vegetation indices (VIs) were extracted from mosaicked aerial images that served as temporal phenomic predictors for southern rust scored in the field and senescence as scored using UAS-acquired mosaic images. Temporal best linear unbiased predictors (TBLUPs) were calculated using a nested model that treated hybrid performance as nested within flights in terms of rust and senescence. All eight machine learning regressions tested (ridge, lasso, elastic net, random forest, support vector machine with radial and linear kernels, partial least squares, and k-nearest neighbors) outperformed a general linear model with both higher prediction accuracies (92–98%) and lower root mean squared error (RMSE) for rust and senescence scores (linear model RMSE ranged from 65.8 to 2396.5 across all traits, machine learning regressions RMSE ranged from 0.3 to 17.0). UAS-acquired VIs enabled the discovery of novel early quantitative phenotypic indicators of maize senescence and southern rust before being detectable by expert annotation and revealed positive correlations between grain filling time and yield (0.22 and 0.44 in 2020 and 2021), with practical implications for precision agricultural practices.

## Introduction

Field-based high-throughput phenotyping (FHTP) is an emerging approach that can provide knowledge and decision-making tools to plant breeders, geneticists, agronomists, and producers^1^. Examining temporal phenotypic data of genotypes promotes mapping of interactions between complex traits, changing environments, and genetic backgrounds within spatio-temporal dimensions. Understanding mechanisms of abiotic and biotic stress tolerance and selecting resistant plants are important goals of breeding programs’ improvement but also for fundamental knowledge generation about pathology, genetics, and overall biology. Implementing unoccupied aerial systems (UAS, also referred to as drones) into plant breeding and genetics programs permits quantification of complex traits across plant growth stages in segregating populations^2,3^. UAS allow accurate prediction of complex traits in untested genotypes across untested target environments^4^. In this research, temporal phenomic data obtained during multiple growth stages of maize hybrids was captured using a UAS equipped with an RGB sensor to train phenomic models to predict southern rust and senescence, which are important biotic, abiotic, and physiological indicators of plant growth. In addition, different days to senescence values, which were scored using high-resolution drone images belonging to late flights in reproductive stages, were incorporated with flowering times to investigate the grain filling period as well as grain yield in maize.

### Southern rust

Southern rust (*Puccinia polyspora* Underw.) is a biotrophic foliar disease affecting maize (*Zea mays* L.). Since its emergence in Western Africa in 1949, where it caused yield losses of ~ 50%^5^, southern rust epiphytotics were responsible for ~ 80% yield loss in the Philippines^6^, ~ 45–50% yield loss in the Mississippi Valley in the early 1970s^7^, and ~ 40–50% yield loss in China in the late 1990s^8^. In contrast, today Southern rust infections infrequently reach economic threshold losses in Texas to justify fungicide treatment^9^ because of non-conducive weather. Plant breeders face several challenges in combating southern rust, from expert scores being subject to rater variability^10^, significant time commitments required for pathologists to score large fields, to selecting for multiracial pathogen resistant germplasm in which general (horizontal) resistance, often found in tropical germplasm, is favored^11^. To ensure broad resistance, diverse germplasm must be screened repeatedly. HTP for rust can be employed via UAS to study disease, using approaches tailored to each pathogen.

### Senescence

Senescence, the deterioration of biological life, is the final stage of a determinant plant’s developmental program, which in maize and other annual crop plants occurs after completion of the reproductive stage^12^. The senescence process is rooted in an organism’s evolutionary history involving complex signaling and regulatory pathways, many of which are unknown^13^. Extending photosynthetic lifespan and plant development, both relating to leaf senescence, have historically improved yield potential in maize^14–16^. The nutrient ratio of source (supply) to sink (demand) during grain filling is understood to regulate senescence^15^. Senescence is intricately linked with the stay-green phenomenon but is complex, as yield and stay-green are not always positively correlated^17,18^. Because photosynthetic lifespan of leaves plays a crucial role in biomass accumulation in crops^19^, stay-green phenomena and premature leaf senescence can be important metrics observable across time points and plant growth stages. UAS-based temporal remote sensing approaches facilitate elucidation of relationships between metrics such as senescence, stay-green, source to sink ratio, and grain filling at a time scale and resolution not previously possible. The effectiveness of UAS imagery has been demonstrated previously in quantitatively scoring maize senescence with moderately high heritability and robust genetic correlation with grain yield, however these results evaluated senescence only once at the late grain filling stage^20^. Senescence variation of breeding lines can be scored using multiple time points during the late reproductive stages that permit examination of more detailed associations between yield and senescence. The present study affords high temporal resolution in senescence thanks to more time points used for senescence scoring than previous studies, as well as establishes phenomic predictive models based on UAS mosaics from multiple time points. Annotation of senescence scores was performed using orthomosaic images (instead of ground-level scoring) corresponding to two late-season dates in 2020 and four in 2021, and this served as training data for phenomic predictive models. Scoring senescence using UAS images facilitates quantification of senescence for thousands of plots at several time points. This is the first reported incidence of multiple-timepoint senescence annotation in maize using orthomosaic images. Accurate assessment of senescence via UAS images eases elucidation of grain filling time and its relationship to yield by calculating the distance between days to flowering and senescence times.

### Grain filling period

The grain filling period in maize is the duration between when a plant flowers and is fertilized until senescence or black layer formation within the kernels. Earlier work demonstrated that actual filling period duration (AFPD), defined as days from mid-silking to black layer maturity, and effective filling period duration (EFPD), defined as kernel size divided by average dry kernel weight accumulation rate during mid-grain filling period, displayed high correlation between years^21^. In the same study, AFPD and EFPD were both independently found to correlate with yield, supporting the hypothesis that an extended grain filling period may be responsible for increased yield^21^. Grain growth and leaf senescence are the primary phenomena during grain filling^22^. Grain filling is responsible for biomass accumulation of starch, protein, and oil in a linear fashion, with leaf photosynthetic rate decreasing linearly during grain filling^22–24^. Grain filling has a complex relationship with senescence, with Abeledo et al.^22^ finding grain weight more sensitive to reductions in source-sink ratio than senescence. The grain filling period is routinely used to parameterize crop models. Investigation of senescence variation with high throughput temporal data enables dissection of grain filling period that will help characterize candidate maize hybrids for target environments with optimized grain filling period^21^.

The objectives of this study were to (i) use temporal phenomic data generated from multiple drone images to predict southern rust and senescence severity occurring in late reproductive stages in maize; (ii) determine which machine learning regressions are best for predicting unknown genotypes in target environments using temporal phenomic data; (iii) uncover if temporal traits constructing the phenomic data were temporally heritable; and (iv) elucidate whether temporal senescence scored by drone images revealed relationships between the grain filling period and grain yield in maize.

## Materials and methods

### Experimental design

Field experiments were carried out at the Texas AgriLife Experiment Station in Burleson County, Texas in the summer 2020 and 2021 growing seasons. Planting dates were 17 March 2020 and 29 March 2021. In 2020, three trials of interest, denoted as trials 1, 2, and 3, were grown under dryland (without irrigation) conditions while three 2021 trials of interest, trials 4, 5, and 6, were grown using full furrow-irrigated conditions. In trial 1, 104 maize hybrids were grown, 303 were grown in trial 2, and 102 were grown in trial 3. In trial 4, 112 maize hybrids were grown, with 100 hybrids each grown in trials 5 and 6 (Supplementary Data 1). The genetic origins of these hybrids were diverse, but elite, and selected from the Texas A&M maize breeding and genetics program. A randomized complete block design was employed with a range and row grid layout in which two replications (reps) were used with 1040 plots in 2020 and 600 plots in 2021.

Ranges corresponded to horizontal gridlines (lines perpendicular to the tractor rows) and rows corresponded to vertical gridlines (lines parallel to the tractor rows). Each plot had two adjacent rows of the same variety. Ranges were 8 m in length and rows were separated by 0.8 m.

The hybrids used in this study were developed in the Texas A&M University maize program; they comply with relevant institutional, national, and international guidelines and legislation.

### Field-based high throughput phenotyping and image processing

Images were captured using a quadcopter UAS (DJI Phantom 4 Pro v2.0) with a 1-inch 20-megapixel CMOS RGB sensor. Field images (orthomosaics) were created using Agisoft PhotoScan (Agisoft LLC, St. Petersburg, Russia). To create the best quality orthomosaics, 90 percent image overlap was used when meshing. UAS mosaics of sufficient quality were obtained 13 and 17 times for the 2020 and 2021 trials respectively. Flight dates in both calendar format and days after planting (DAP) are in Table 1.

**Table 1:** Flight times in 2020 and 2021.

2020 flight times																
April					May		June				July					
3rd (17)	8th (22)	16th (30)	20th (34)	28th (42)	15th (59)	18th (62)	5th (80)	11th (86)	16th (91)	20th (95)	7th (112)	12th (117)				

2021 flight times																
April	May					June					July					
27th (29)	6th (38)	12th (44)	18th (50)	27th (59)	30th (62)	2nd (65)	6th (69)	13th (76)	15th (78)	21st (84)	1st (94)	10th (103)	16th (109)	23rd (116)	27th (120)	29th (122)

Flight times were given as calendar dates and corresponding days after planting (DAP) in parentheses.

### Data extraction from remotely sensed images

Populations of interest were assessed using QGIS geospatial data software (QGIS Development Team, 2021). Data extraction was carried out in RStudio (RStudio Team, 2021). After orthomosaic cropping, a plot-labeled grid file (shapefile) was created and overlaid in QGIS using *UAStools* such that all plots were labeled according to range, row, and hybrid based on field maps for the 2020 and 2021 growing seasons^25^. Vegetation indices (VIs) were extracted using the *FIELDimageR* package^26^. An overview of the VI extraction protocol used is listed here: (1) to remove noise before extraction, soil was cropped out of the orthomosaic using the *FIELDimageR::fieldmask()* function, (2) VIs were defined in *FIELDimageR::fieldindex()* function, (3) extraction of VIs was performed within boundaries defined by the shapefile using the *FIELDimageR::fieldInfo()* function. All 36 VIs in this study have been provided in Supplementary Table 1 alongside respective references.

### Temporal phenotypic data

A fully random fit model was constructed in *lme4* in R with the restricted maximum likelihood approach used for predicting variance component estimation and temporal best linear unbiased predictors (TBLUPs) for each maize hybrid as explained in Adak et al.^4^. Range, row, and replicate were also treated as nested model terms to account for temporal field spatial variation.

A nested model design was used to predict TBLUPs of VIs for both 2020 and 2021, denoted by Eq. (1) below:

1 $${Y}_{ijklm}=\mu +{T}_{i}+{H}_{i\left(j\right)}+{Range}_{i\left(k\right)}+{Row}_{i\left(l\right)}+{Rep}_{i\left(m\right)}+{\varepsilon }_{ijklm}$$

*Y* signifies each VI observation of each maize hybrid at each time point *i*, given as DAP; *μ* signifies the grand mean; *T* signifies the effect of each flight date *i* in DAP (*i* in 2020: 17, 22…117; *i* in 2021: 29, 38…122); *H* signifies the effect of each maize hybrid *j* within each flight date *i*; *Range* signifies the effect of each range *k* within each flight date *i*; *Row* signifies the effect of each row *l* within each flight date *i*; *Rep* signifies the effect of each replication *m* within each flight date *i*; and *ε* (σ^2^_*error*_) signifies the combined error accounting for residuals of all aforementioned variance components.

Temporal repeatability was calculated according to Eq. (2):

2 $${\text{Temporal}}\;{\text{repeatability}} = \frac{{H_{{i\left( j \right)}} }}{{H_{{i\left( j \right)}} + \frac{{\varepsilon _{{ijklm}} }}{{{\text{no}}{\text{.}}\; {\text{of}}\; {\text{reps}}}}}}$$

A phenomic data matrix was created by merging TBLUPs of all vegetation indices belonging to each maize hybrid for 2020 and 2021. Phenomic data is attached as Supplementary Data 1 in this study. In the phenomic data of both years, each column header included VI and days after planting (VI_DAP).

### Predicted variables

**Figure 1:**
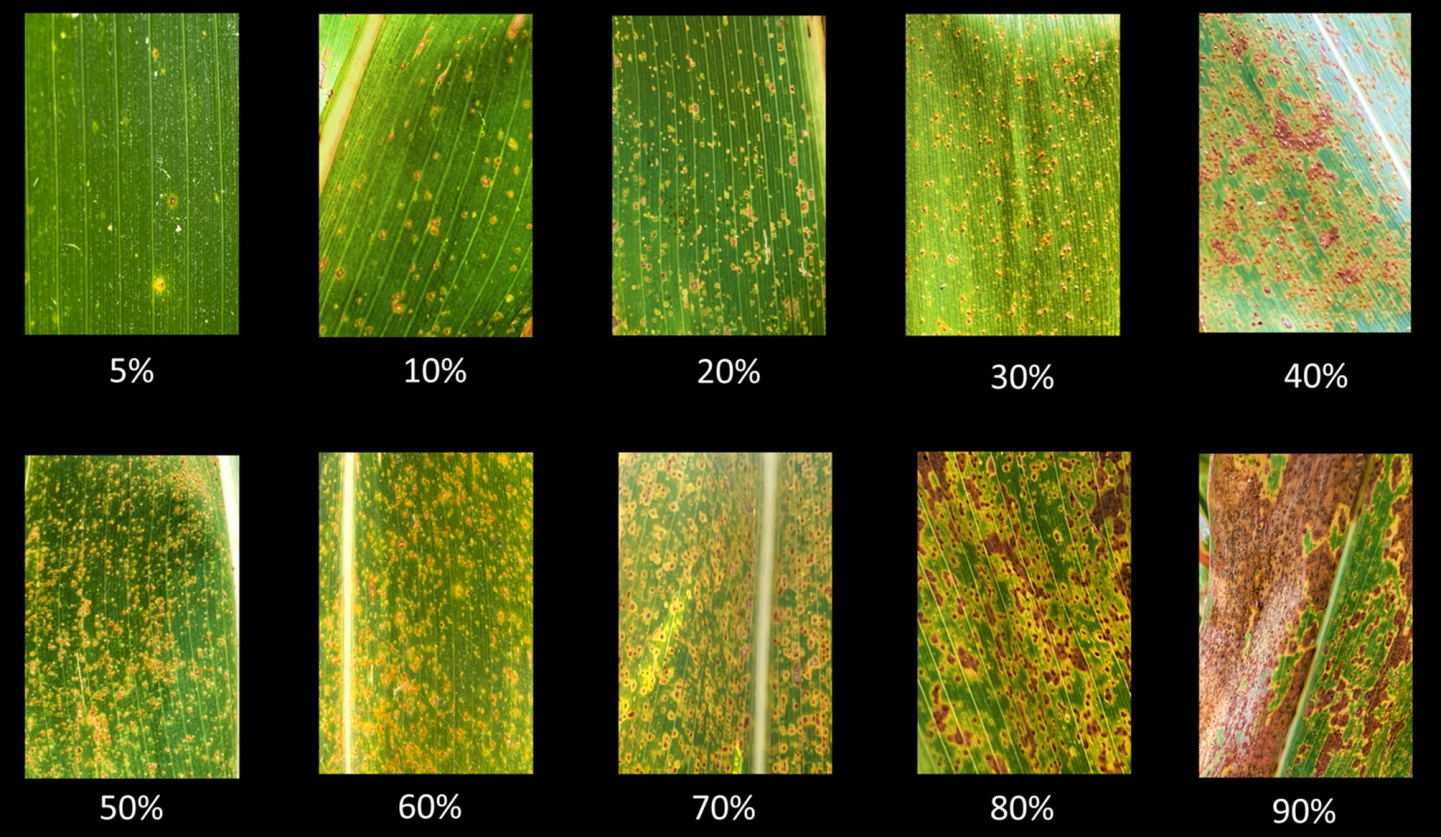
Visual scoring system for southern rust based on percentage leaf area covered by rust pustules.

This study was conducted to predict southern rust severity and senescence progression. Rust was scored in the field on 26 and 27 July 2021 using the three trials (4, 5 and 6) in 2021. Rust was scored using a 0 to 100 scale, with 0 representing 0% leaf coverage and 100 representing complete leaf coverage of rust pustules. An approximate visual guide to percentages is detailed in Fig. 1.

**Figure 2:**
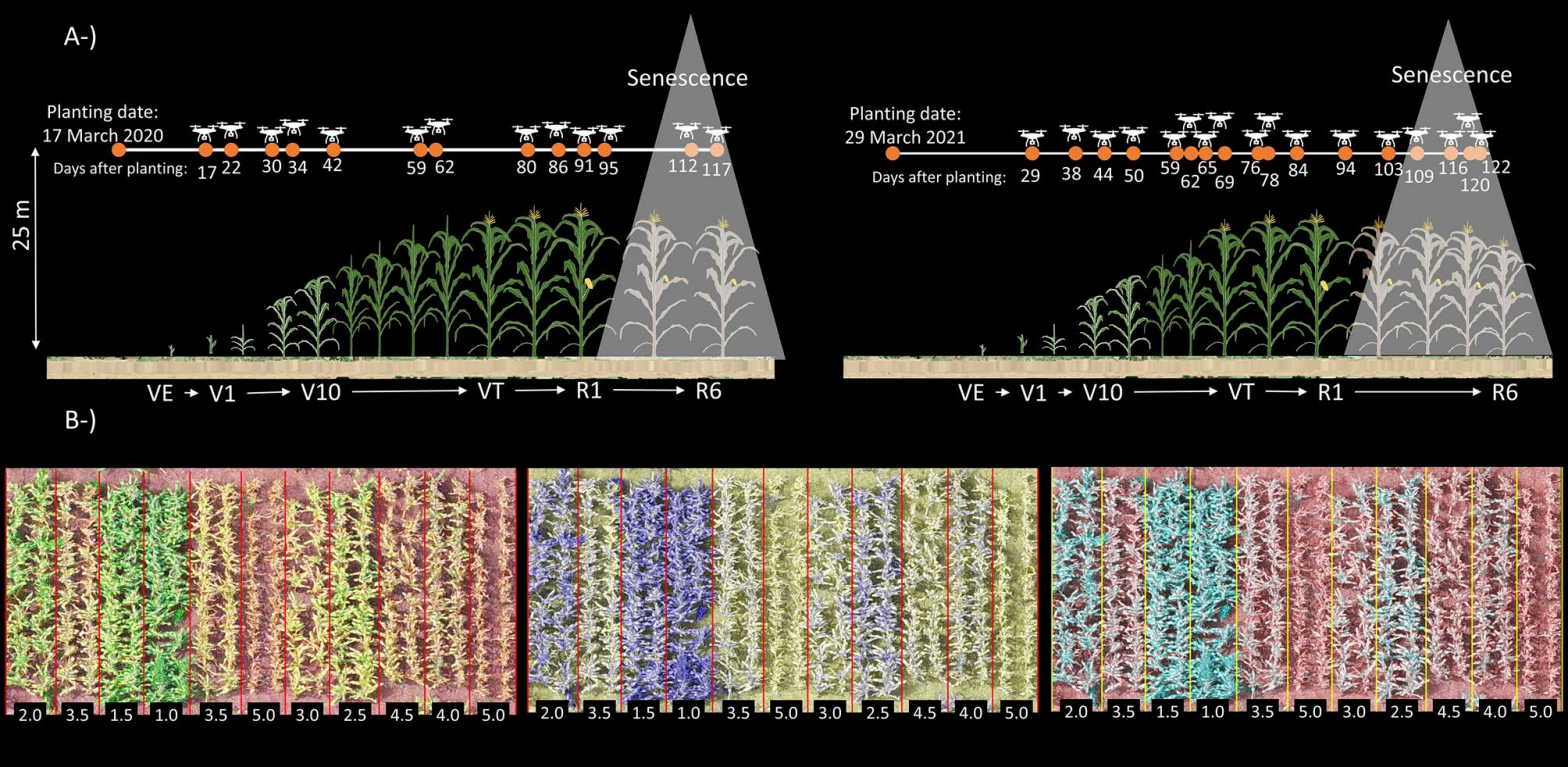
(**A**) The number flight times used in 2020 (left) and in 2021 (right); senescence scored using the orthomosaics of latest four flights in 2021 and two flights in 2020. (**B**) Variation in senescence and illustration of senescence scores on a scale of 0 to 5 (numbers in black boxes) among different maize row plots (each is plot is two rows, outlined by red or yellow rectangles). Three band channels in orthomosaics were set in QGIS as red–green–blue, red–red–green, and red-green-green from left to right respectively and resulted in three different illustrations in highlighting the row plots with different senescence scores.

Senescence scores were annotated twice for 2020 using two orthomosaics corresponding to the last two flight dates in 2020 (7 and 12 July: 112 and 117 DAP) and for 2021 using the last four orthomosaics generated in 2021 (16, 23, 27, and 29 July: 109, 116, 120 and 122 DAP), made possible by high-resolution afforded by low flight elevation (25 m). After shapefile overlay in QGIS, senescence scores were annotated visually using a 0 to 5 scale based on percentage of tissue death, with 0 representing no signs of senescence and 5 representing complete senescence (Fig. 2).

For hybrid values of senescence predicted as variables in phenomic prediction models, Eq. (1) was used by replacing the flight component with the time component containing two and four dates of scoring senescence for each trial in 2020 and 2021 respectively. Similarly, to predict the hybrid values of Southern rust to use as predicted variables in phenomic prediction models in 2021, Eq. (1) was run without the flight component, for rust, flowering times (days to anthesis and silking; DTA and DTS), three types of terminal height measurements (from ground level to tip of tassel, flag leaf collar, and shank of first ear; PHT, FHT, EHT respectively) and yield (t/ha) to predict the hybrid values for each hybrid in each trial in each year.

Grain filling time was calculated as days between DTA and days to senescence, as estimated by a linear model. In order to calculate the days to senescence, (i) a linear model of senescence scores (Y axis) over dates (X axis: in unit of DAP) used for senescence scoring for each hybrid in each trial in each year was fit; (ii) linear models of each hybrid were then constructed and used to predict the three different senescence times where senescence scores for each line were set equal to 3, 4 or 5 were used as response; (iii) lastly, DTA of each hybrid was subtracted from the three different senescence times of each hybrid to calculate three different grain fill times indicating by grain_fill(3), grain_fill(4) and grain_fill(5). These predicted days to senescence scores were given in Supplementary Data 1.

Temporal repeatability was calculated for senescence and repeatability was calculated for rust, flowering times, heights, and yield using Eq. (2). Correlation coefficients were calculated among flowering times, plant heights, senescence scores, rust, yield, and grain filling times in both years using *ggcorrplot* package in R.

### Phenomic prediction models

In the phenomic prediction pipeline, rust, two senescence scores belonging to 2020, and four senescence sores in 2021 were predicted using the phenomic data of 2020 and 2021. Phenomic prediction, where a machine learning model uses patterns assembled from training data where each hybrid’s information is provided alongside predictors to estimate performance of untested hybrids, was conducted using phenomic data from 2020 and 2021 with eight machine learning algorithms in the *Caret* package in R. Beginning with an iterative procedure, data split was partitioned as 70 and 30 percent training and test respectively in each of 500 bootstraps. Second, phenomic prediction accuracies were obtained between true breeding values (TBVs) and phenotypically estimated breeding values (PEBVs) in each bootstrap. As a result, 500 prediction accuracies were obtained for each phenomic prediction model, and prediction accuracies were evaluated in contrasts using student’s *t*-tests.

Within the *caret::train()* function, eight machine learning regression models used for phenomic predictions were defined as follows: method in *caret::train()* function was set as “*lm*” for linear regression, “*glmnet*” for ridge, lasso, and elastic net, “*rf*” was set for random forest regression, “*svmLinear*” was set for support vector machine regression with linear kernel, “*svmR*” was set for support vector machine regression with radial kernel, “*pls*” was set for partial least squares regression, and “*knn*” was set for k-nearest neighbors regression. Alpha was set at 0 for ridge regression, 1 for lasso, searched between 0–1 for elastic net using the *expand.grid()* function. The code used in this analysis is viewable at (https://github.com/alperadak/phenomic-prediction-/blob/main/Phenomic%20prediction). *Ntree* was set at 1000, while *mtry* was searched using the *expand.grid()* function to find optimal *mtry* number in the random forest regression. To find the optimal *cost* value (with the lowest root mean squared error), *expand.grid()* was used in in support vector machine regressions. *Tunelength* was set at 100 in both partial least square regression and k-nearest neighbors algorithm to find the optimal number of principal components and number of *k*, respectively. Variable importance scores were obtained using the *Lasso* algorithm for each predicted variable in both years.

## Results

### Variance explained by nested design for temporal phenotype

**Figure 3:**
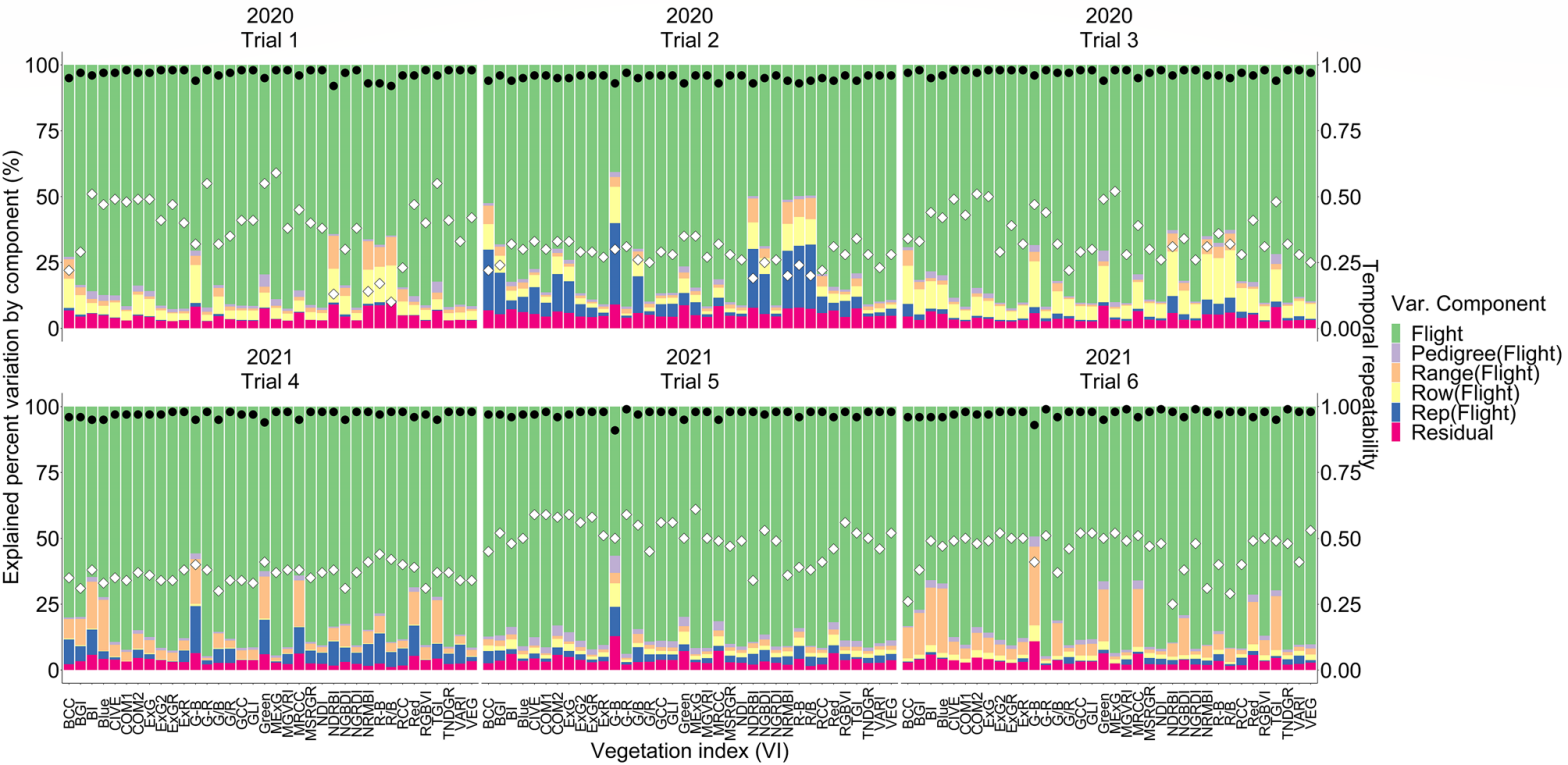
The explained percent variation by each component in nested design [Eq. (1)] was illustrated by a stacked bar plot. Left Y axis shows the explained percent variation by each component in nested design, the X axis shows the VIs for each trial and year. Right Y axis shows the scale for R-squared and temporal repeatability. Black circles indicate R-squared and white diamonds indicate temporal repeatability.

The flight component in the nested design explained the highest percent of experimental variation for all VIs in both years, changing between ~ 41 and 95 percent (Fig. 3); temporal genotypic variance denoted by hybrid nested within flight in the nested design explained percent variation between ~ 0.5 and 7 depending on the VIs (Fig. 3). Temporal repeatability was calculated between ~ 0.1 to 0.6 depending on the VIs in both years (Fig. 3).

**Figure 4:**
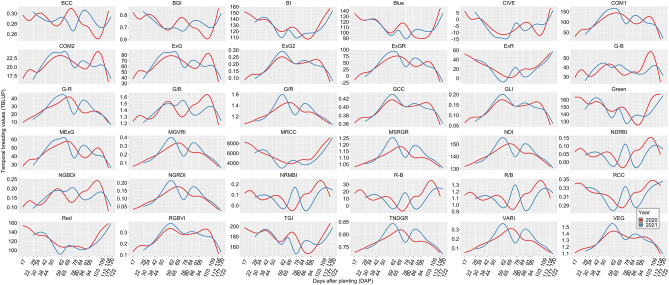
The smoothed conditional mean of temporal breeding value of each hybrid for each VI belonging to 2020 (red lines) and 2021 (blue lines) predicted by hybrid nested within flight component in nested design [Eq. (1)].

**Figure 5:**
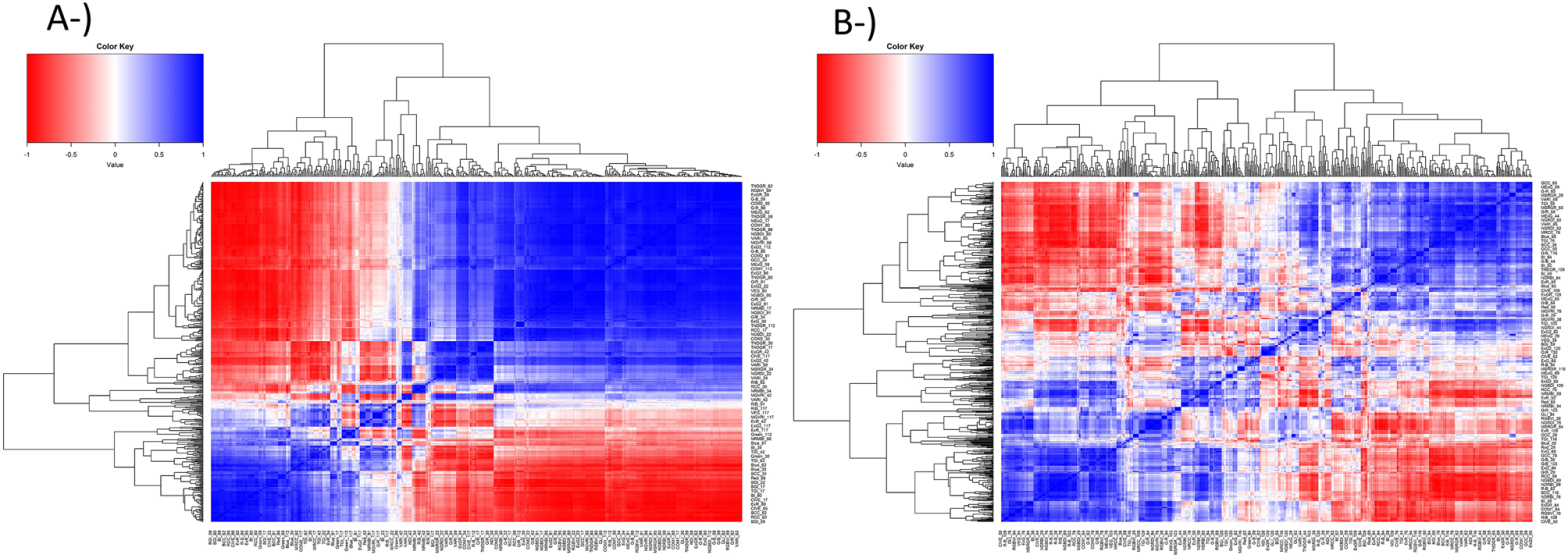
Pearson correlation coefficients for (**A**) 2020 and (**B**) 2021 phenomic data.

Results of hybrid nested within flight component in nested design [Eq. (1)] were visualized to reveal the temporal breeding values of each VI belonging to maize hybrids in both years. Temporal resolution revealed the different temporal patterns for different VIs across flight times where distinct physiological plant development stages were found to have varying temporal values. For instance, temporal breeding values at plant emergence, flowering times, and the conclusion of the reproductive stage usually had the most extreme VI values (Fig. 4). Correlation coefficients of phenomic data between genotypes within both 2020 and 2021 were found to vary significantly; correlation coefficients were found to be strongest within the same growth stages and weaker between different growth stages, notably before and after flowering (Fig. 5). In other words, the time factor was found to be more important than the VI factors to obtain diverse correlation coefficients in phenomic data of both years.

**Figure 6:**
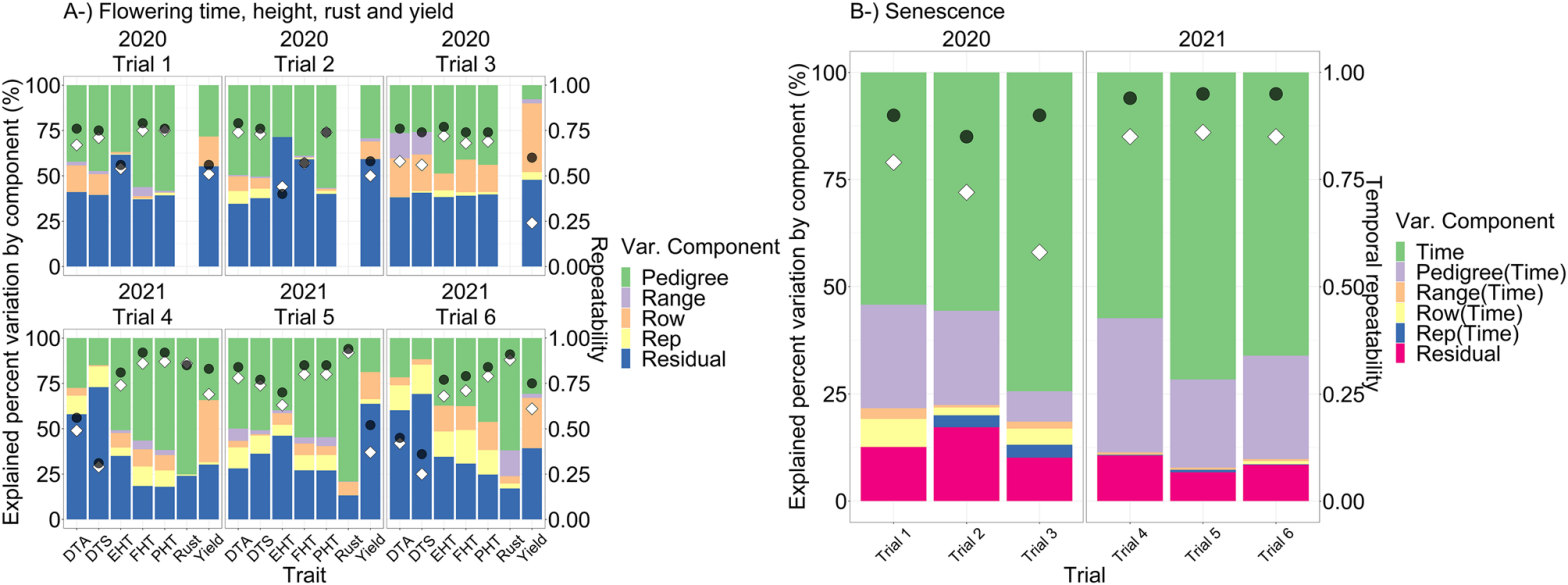
(**A**) Explained percent variation for flowering times (days to anthesis and silking: DTA and DTS), three different plant heights (plant heights from ground to tip of tassel: PHT, to collar of flag leaf: FHT and to shank of first ear: EHT) in each trial in both years based on left Y axis. (**B**) Explained percent variation for senescence for each trial in each year. Black circles indicate the R-squared value and white diamonds indicate the repeatability for flowering times, heights, rust, yield, and temporal repeatability for senescence based on right Y axis.

**Figure 7:**
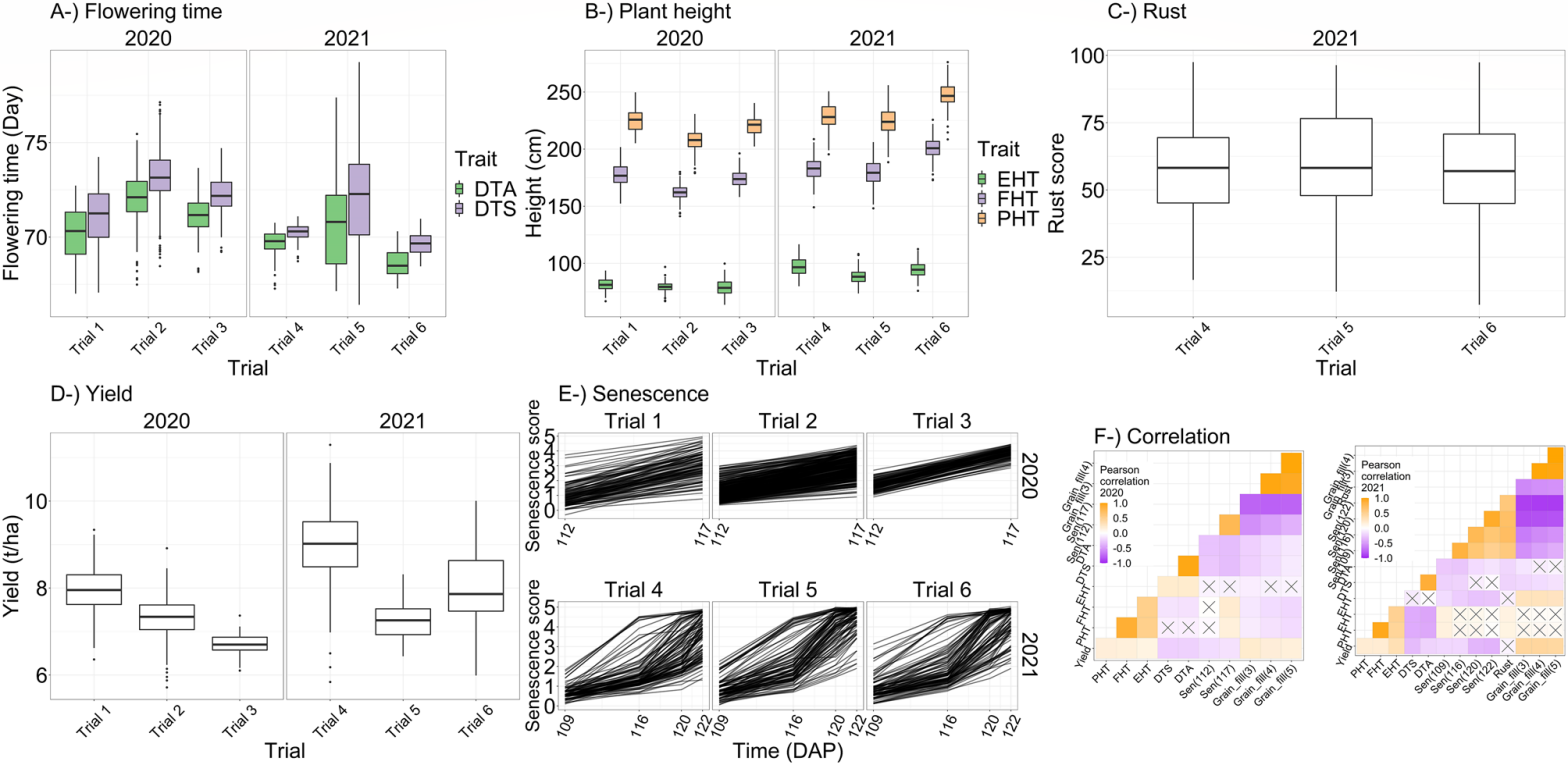
Predicted hybrid values of (**A**) flowering times (days to anthesis and silking: DTA and DTS), (**B**) three different plant heights (plant heights from ground to tip of tassel: PHT, to collar of flag leaf: FHT, and to shank of first ear: EHT), (**C**) rust, (**D**) yield, and (**E**) senescence in each trial in both years. (**F**) Pearson correlation coefficients were given that were calculated among all predicted traits including three different grain fill traits in each year.

Temporal repeatability was calculated between 0.6 and 0.9 for senescence depending on the trials in both years (Fig. 6). Repeatability was calculated between 0.3 and 0.8 for flowering times, between 0.4 and 0.9 for the three different height measures, between 0.2 and 0.7 for yield, and ~ 0.9 for rust depending on trials and years (Fig. 6). Predicted hybrid values of flowering times, plant heights, rust, yield, and senescence were given in Fig. 7 for each trial in both years. Correlation coefficients between grain filling times and yield were found to be up to 0.22 and 0.44 in 2020 and 2021 respectively (Fig. 7), which were higher than any correlations between yield and any other traits, including the flowering and senescence estimates making up the grain filling calculation.

### Temporal correlation between senescence/rust and temporal phenotype of vegetation indices

**Figure 8:**
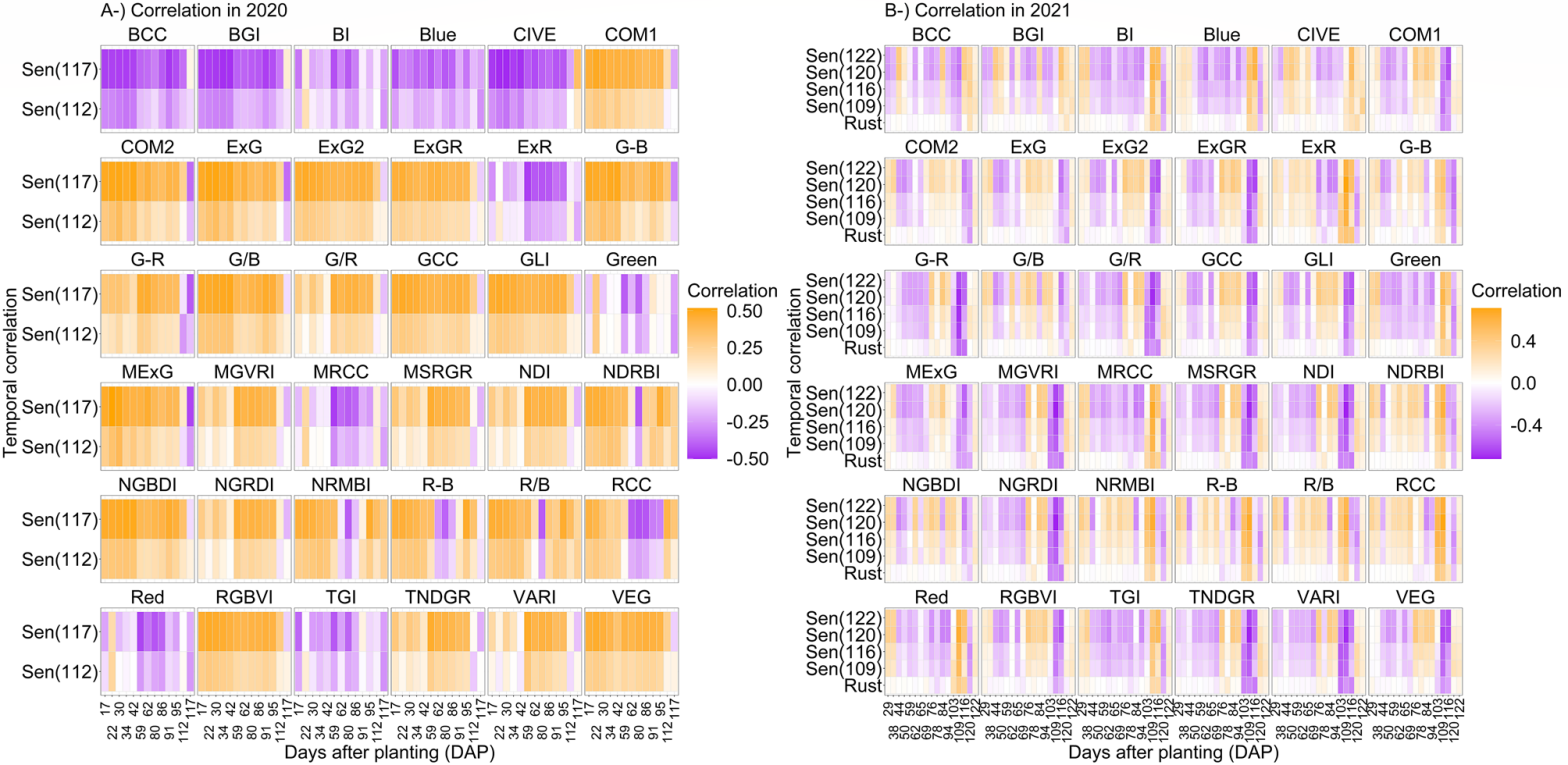
Temporal correlations between predicted variables (two and four senescence scores in 2020 and 2021 respectively, and rust in 2021) and temporal phenotype of each VI belonging to 2020 and 2021 phenotype data. (**A**) and (**B**) represent 2020 and 2021 temporal correlations, respectively.

Temporal correlations were calculated between each of the primary predicted variables (senescence in 2020 and 2021 and rust score in 2021) and temporal phenotypes of each VI belonging to 2020 and 2021 phenomic data (Fig. 8). Correlation coefficients varied (0.71 for 2020 and 0.72 for 2021); however, most of the temporal correlations were found to change between − 0.5 and 0.5. In 2020, temporal correlations were found to be more stable than those of 2021. Temporal correlations followed consistent trajectories across two flight times in 2020. In contrast, temporal correlation in 2021 appeared more sensitive to different growth stages. For instance, early- and mid-vegetation growth stages were found to have opposite temporal correlations for the four senescence dates in 2021 (Fig. 8).

### Assessment of phenomic prediction models

**Figure 9:**
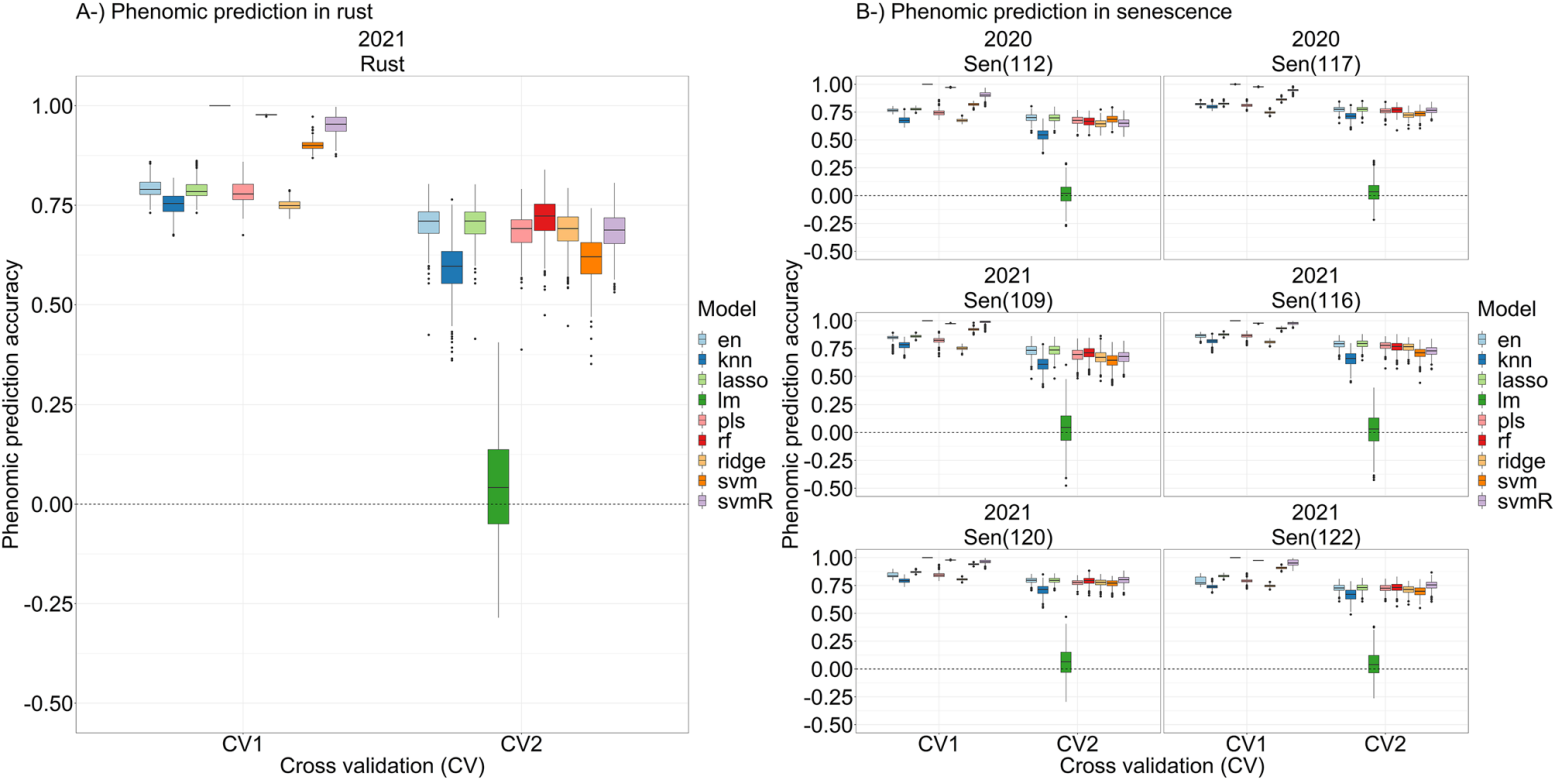
Phenomic prediction abilities of eight machine learning algorithms for (**A**) rust, and (**B**) two and four different senescence scores in 2020 and 2021. Y axes in both (**A**) and (**B**) represent phenomic prediction accuracy; X axes correspond to cross validation where phenomic prediction accuracy was calculated over training data (CV1), and test data (CV2). In all cases, linear model (lm) displayed lowest prediction accuracy.

A student’s *t*-test was applied to prediction accuracies of each model, which incorporated all remotely sensed variables, to compare their means in predicting scores belonging to two senescence dates in 2020 and four senescence dates and one rust date in 2021. Connecting letter reports, which revealed potential statistical differences between prediction accuracy means belonging to each model, are given in Supplementary Table 2. Elastic net and lasso displayed the highest prediction accuracies (~ 0.70 and ~ 0.77 respectively) for senescence scored on 7 July 2020 (112 DAP) and 12 July 2020 (117 DAP) (Fig. 8). Similarly, elastic net and lasso had the highest prediction accuracies (~ 0.73, ~ 0.79, ~ 0.80 and ~ 0.73) for senescence scored on 16 July 2021 (109 DAP), 23 July 2021 (116 DAP), 27 July 2021 (120 DAP), and 29 July 2021 (122 DAP) (Fig. 8). In addition, support vector machine regression with radial kernel had the highest prediction accuracies (~ 0.80 and ~ 0.75 respectively) for senescence on 27 July 2021 (120 DAP) and 29 July 2021 (122 DAP) (Fig. 8). For rust measurements taken in 2021, random forest regression performed best with a prediction accuracy of ~ 0.72, followed by elastic net and lasso regressions with prediction accuracies of ~ 0.70 (Fig. 9). The general linear model was outperformed by all eight machine learning regressions in each case. Mean phenomic prediction accuracies across all predicted traits for each model are provided in Supplementary Data 3. The linear model displayed the highest RMSE values for all predicted traits, ranging between 65.8 and 2396.5. In contrast, all machine learning regressions across all predicted traits ranged between 0.328 to 17.0 (Supplementary Figs. 1 and 2; averages of all RMSE values from each predicted trait and model’s 500 bootstraps are in Supplementary Data 2). Variation in prediction accuracies among machine learning models is a primarily a product of each model’s tolerance for nonlinearity, outliers, and collinearity, amount of training data required, and whether the model is parametric or nonparametric. The usefulness of each model is dependent on the structure of the dataset to which it is applied.

### Variable importance scores

**Figure 10:**
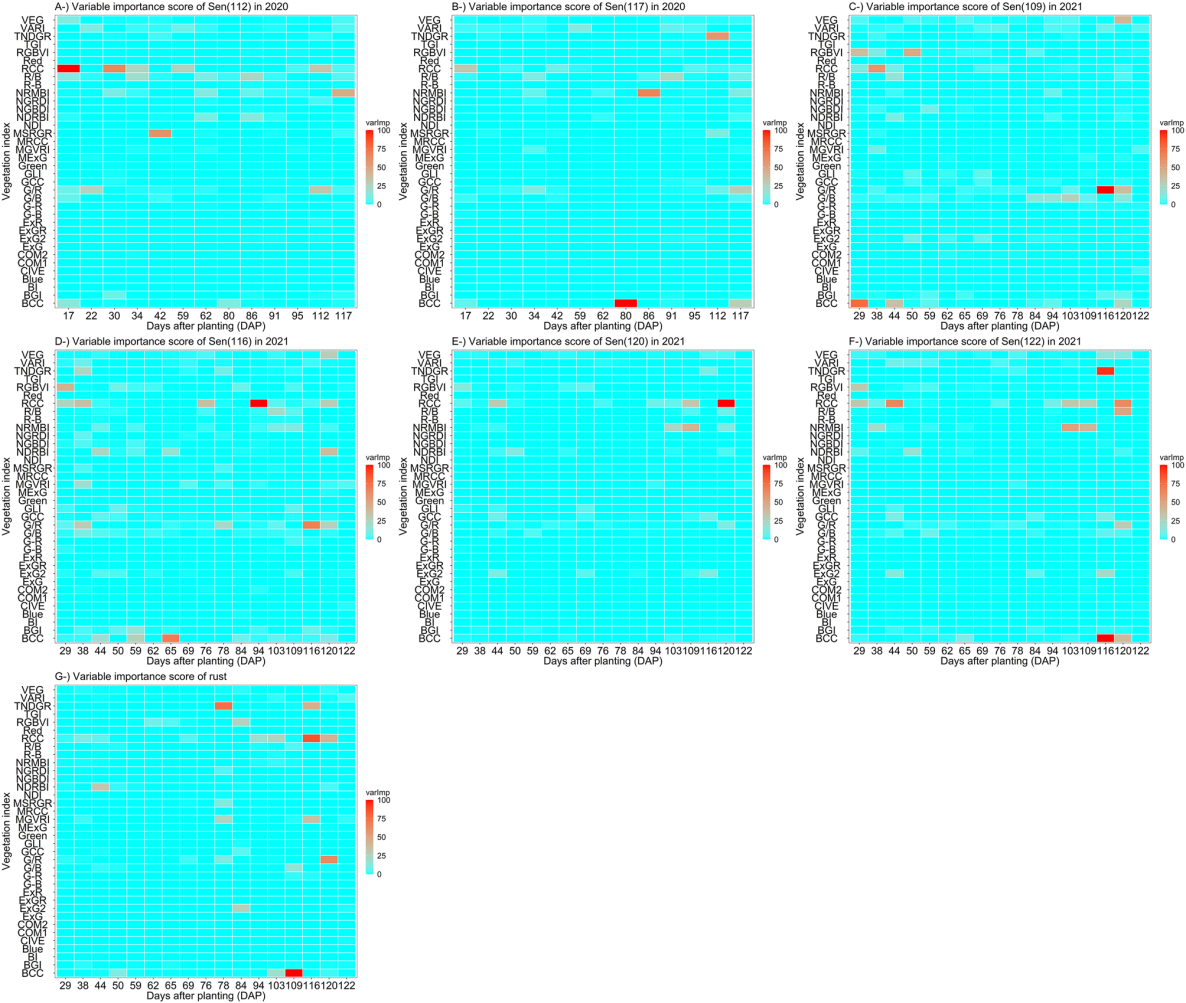
Variable importance scores (*varImp*) scores of 2020 and 2021 phenomic data for two and four senescence scores in 2020 and 2021 respectively, and rust in 2021 obtained by lasso algorithm. Each plot denotes variable importance scores according to the following naming: (**A**) senescence at 112 days after planting (DAP) in 2020; (**B**) senescence at 117 DAP in 2020; (**C**) senescence at 109 DAP in 2021; (**D**) senescence at 116 DAP in 2021; senescence at 120 DAP in 2021; senescence at 122 DAP in 2021; rust in 2021.

Variable importance scores (*varImp*) were generated by the lasso algorithm. Lasso was selected due to its tolerance for many predictors that exert minimal influence on the target variable. Figure 10 reveals important time (in DAP) and VI combinations that uncover early time indicator(s) of rust and days to senescence and before rust and senescence physiologically appear. RCC was nominated (based on *varImp* scores) as one of the critical vegetation indices for rust and days to senescence; its phenotypic values belonging to early vegetation stages (i.e., 17 and 34 DAP in 2020, and 38 and 44 DAP in 2021) were found to be an important early phenotypic indicator. RCC had also the highest *varImp* during days to senescence (Fig. 10). Importantly, RCC temporal phenotype values had mostly negative correlations with all senescence scores in both years during vegetation growth stages, whereas temporal correlations transitioned quickly into the highest positive correlation values when senescence started (Fig. 8). Other important VI/time combinations were illustrated in Fig. 10 and their relationships with predicted variables were given in Fig. 8.

## Discussion

Vegetation indices (VIs) are ratios of light reflectance wavelengths that allow for the quantitative evaluation of growth, vitality, and other morphological features in terrestrial vegetation. Early attempts at obtaining VIs via remote sensing date back to the origins of the precision agriculture movement^27,28^, with the advent of remote sensing traceable to the 1960s^29^. High temporal resolution of VIs was achieved in this study through analysis of 13 and 17 flights during 2020 and 2021, respectively (Table 1; Fig. 4). Within each growth stage (before and after flowering), correlation coefficients of phenomic data were stronger versus between different stages, signifying the temporal component of UAS-acquired phenomic data was more important than the VIs themselves (Fig. 5). Relatively high temporal repeatability values for senescence (0.6 to 0.9) may be attributed to the capture of precise temporal senescence variation of diverse maize hybrids through visual scoring using high-resolution and high dimension orthomosaics. More importantly, temporal scoring of senescence enabled the quantification of differences in senescence acceleration of hybrids over time, which has been disregarded in ground-level scoring due to arduousness of manual annotation, low scaling, and resolution, as well as lack of temporal observations. Temporal scoring of senescence during the reproductive growth stages can be used as a novel trait that can be associated with earlier growth stages (e.g., flowering stages) in maize to dissect the grain filling period. Using temporal senescence dates as end points for grain fill, correlation coefficients were determined between grain filling time and yield (0.22 and 0.44 in 2020 and 2021, respectively) that provided insights into the relationship between senescence (in DAP) and yield. Extending grain filling time is recognized as an important component of both yield and yield under stress^21^; this applies not only to maize but to other annual row crops like closely related sorghum^30^. UAS estimation of temporal senescence scores is a new tool to examine the grain filling period that might be a new selection criterium for plant breeders to select maize hybrids that was previously impractical. This will additionally allow plant physiologists and biologists to better understand plant developmental processes, specifically at end-of-life.

### Machine learning in phenomic predictive models

Automatic recognition of patterns from example data that convey practical significance is the objective of machine learning in data science^31^. Deep learning is a subset of the broader category of machine learning. Shallow machine learning approaches such as support vector machine, *k*-nearest neighbors, decision tree, and others seek to assess problem-specific example data in an iterative manner, promoting pattern recognition and reproducibility of reliable decisions^31,32^. Shallow approaches require handcrafted feature extraction (which in this study was explicit outlining of each plot preceding vegetation index extraction), followed by automated model building as specified by the algorithm used^32,33^. Deep learning approaches employ nested network architectures comprised of neurons organized in various layers. Raw data are fed to an input layer and an output layer generates the desired outcome^32,33^ (i.e., classification, prediction, etc.). As implicated by the name and not dissimilar to its creators, a “neural” network must study; to decipher relationships between genotype, phenotype, and other biological outcomes, it must be granted access to training data in which those relationships exist and have been previously characterized^34^. The lack of necessity for explicit feature outlining with deep learning approaches makes such approaches desirable for high-dimensional biological data, however this often demands access to significant computational resources. In the context of agricultural research, some groups may not have access to high-performance workstations or university supercomputing clusters that (at the time of writing) are required for deep learning analysis of spatio-temporal agricultural datasets to be conducted within a reasonable timeframe. In addition, deep learning approaches are not always interpretable or explainable, making it difficult to understand the underlying biological phenomenon as well as to trust the approach to be meaningful. Often regarded as “black boxes,” an ongoing area of investigation with deep learning models is to address deficits in model transparency and functionality^35^. This study implemented shallow machine learning approaches due to rapid turnaround of interpretable data and the relatively lower resources needed for these analysis methods.

Phenomic predictive models with the highest accuracies in this investigation were machine learning-based, each outperforming the linear model. Statistically significant differences between model accuracies were found, however they were relatively minor, with all models except for the general linear model performing similarly for both rust and senescence predictions, (Fig. 9). This study builds on previous work that has demonstrated the effectiveness of machine learning and deep neural networks in yield prediction^4,36,37^, disease and pest detection^38,39^, biomass and nitrogen content^40^, and others (for a comprehensive list, see Jung et al.^41^). Using the *lasso* algorithm, variable importance scores revealed important early phenotypic indicators, with the red chromatic coordinate index (RCC^42^) consistently emerging as a predictive early marker for rust and senescence (Fig. 10). The RCC index has previously demonstrated usefulness in tracking the maximum intensity of autumn colors in deciduous forests^43^, the same reflectance bands may also characterize aging in maize leaves. Liu et al.^44^ established RCC as a reliable indicator of anthocyanin reflectance in end-of-season forest canopy photosynthesis and reported it peaking early in the season not in relation to soil exposure in the image dataset following spring snowmelt, asserting that RCC’s peak early in the season was responding to the canopy. This bolsters findings in Fig. 10 revealing RCC as an early indicator of senescence. These previous findings also lessen the likelihood that exposed soil appeared as an artifact in the variable importance of RCC. Future studies might focus on characterization of the molecular phenomena giving rise to reflectance bands being captured by RCC. Blue chromatic coordinate index (BCC), TNDGR, and RCC emerged as mid- and late-season indices that were more important for predicting southern rust. Identification of robust indicators of disease progression and senescence in cereal crops, particularly at time points occurring before flowering (Fig. 10), has implications for precision agricultural practices provided future studies can validate early-season predictions.

### High throughput phenotyping: implications for plant breeders and geneticists

The efficacy of using HTP prediction of agronomic traits has been demonstrated in other crop breeding programs, including tomato yield^45^, crop cover biomass in legumes^46^, soybean yield^47^, wheat grain yield^48,49^, and for height, yield, flowering time, and kernel dimensions in maize^4,50–52^. Montesinos-López et al.^53^ revealed a growing role for hyperspectral image data in maize yield prediction, finding that increased prediction accuracy was achieved through simultaneous analysis of all hyperspectral bands versus using nine VIs alone. This is in consonance with the findings of Aguate et al.^54^ that hyperspectral images can lead to generation of models with better predictive performance for grain yield as opposed to using VIs, specifically when using Bayesian shrinkage and variable selection methods when combining temporally collected images. However, the present study highlights the capability of a simple RGB camera on a UAS to provide spatio-temporal data able to train machine learning models to predict important phenomena such as southern rust progression and senescence. Similarly, using conventional handheld digital camera images at the canopy level, Vergara-Diaz et al. showed that RGB indices can be used in prediction of grain yield and yield loss in wheat infected with yellow rust, also citing its empirical advantages over subjective ground-based scoring methodologies^55^. Zhou et al. found RGB camera-derived color parameters such as hue, green fraction, and greener fraction, dubbed as picture-derived VIs, represented genotypic variability of yellow rust infected wheat^56^. Though they capture less spectral information, RGB images remain a viable foundation for phenomics studies. It is also important to note that higher temporal and image resolution were provided in this study by lower flight altitude (25 m) and higher number of flights (13 and 17 time points) to generate the high throughput phenomic data that have been disregarded so far by most of the current literatures. Higher temporal and image resolutions were proposed to be important in predicting complex traits with higher accuracies, followed by the number of wavelengths and sensors^57,58^. Moreover, temporal resolution in high throughput phenomic data is required to dissect the growth stages in greater detail, particularly when the determination of critical time points as selection criteria are a goal in plant breeding programs.

It was highly surprising that senescence, an end-of-life measure, had some predictability as early as 17 days after planting (Fig. 8); we believe this is likely due to features of predictions from relatedness, not a shared physiological cause. This study confirmed previous findings that UAS HTP can facilitate understanding of genotypic response to disease in a manner complementary to traditional disease phenotyping methods^59,60^, as well as to replace subjective visual assessment of senescence with quantitative, phenotyping-based screens of large breeding populations^20^. Preliminary findings in this study highlight the potential for grain filling period to serve as a predictor of yield. This would be a logical trait to extract for temporal UAS data both on its own, or as a component of phenomic selection. UAS HTP may remove the need for labor intensive measures of grain filling, however this trait is collected at the end of the season and may not practically speed up the breeding cycle.

HTP also has applications in supplementing established genetic techniques. Xavier et al.^61^ tracked soybean canopy development from stage V2 to R5, and after detecting a QTL responsible for yield increase by genome-wide association study (GWAS), determined fast canopy coverage is an inexpensive early season trait that has value for programs focused on yield maximization. In maize, UAS flights conducted at V5, V12, V15, and R stages supplied the necessary temporal resolution to reveal eight QTLs simultaneously controlling plant height and growth rate at different maize life cycle stages^62^. In a large-scale QTL analysis, phenotypic data across 16 maize developmental stages contributed to the uncovering of three QTL hotspots^63^. Pauli et al.^64^ supplemented a temporal study of QTL in cotton stress response with HTP tracking canopy temperature, reflectance, and height, revealing a temporal dimension to QTL expression. The use of VIs and canopy temperature as predictors were shown to increase model accuracy of genomic and hybrid model accuracies for wheat grain yield^65^. Sandhu et al.^66^ suggested using spectral information as a secondary trait in genomic prediction provided better prediction accuracy for grain protein content. Galán et al.^67^ argue that genomic models incorporate genetic relationships between untested candidates and those with known genotypic and phenotypic information, and their study revealed hyperspectral reflectance-derived (HBLUP) relationship matrices (i.e., HTP data) were less prone to genetic relatedness and trait heritability, whereas more highly heritable traits were better predicted by genomic (GBLUP) relationship matrices. This finding reveals a dichotomy and interplay between applications for phenomic and genomic predictive models. Phenomic and genomic predictions both excel in specific experimental environments; the ideal hybrid predictive model will utilize facets of one where the other has limited predictive power.

### Challenges, proposed solutions, and future directions

The quality of aerial imagery, as with all photography, is subject to lens distortion, white balance, aperture size, shutter speed, and solar angle. However, workflows have already been developed that employ pre-processing techniques to correct many of these issues^68^. Manual control of UAS sensor settings may afford less unexplained model variance in future studies. However, it is also important to note that VI’s, which use a ratio of reflectance bands, tend to associate more to critical variation than raw values^69^ as observed in correlation coefficients with predicted variables in Fig. 8 with raw bands having lower correlations than VIs. Similar studies in the future may benefit from concurrent multispectral and RGB flights to determine if other wavelengths might better capture progression of disease and senescence with less unexplained model error. Zhang et al.^39^ found that RGB-based color features were less effective than multispectral features in quantitative detection of rice sheath blight, but that color space transformation can lend to improved disease severity quantification by reducing effects of brightness differences as well as strengthening saturation, hue, and other metrics, like Fig. 2 above. However, given the higher throughput temporal nature of the present study, RGB-based color features were found to yield high phenomic prediction accuracies (Fig. 9) and correlated well with ground truth data (Fig. 8). This is likely due to a “quantity over quality” phenomenon in which strong signal emerges amid capture of a high volume of spatio-temporal data, even from less complex wavelengths such as those in the visible RGB spectrum, and where minor camera errors may be present between flights.

As the magnitude of data collected places strain on computational power, memory, and storage, another issue arises in the form of data wrangling and processing. Future studies might investigate the intersection between meaningfulness of results, spatio-temporal resolution, volume of data collected, and cost–benefit analysis of the aforementioned. In predicting soybean maturity date, Volpato et al.^70^ found after testing flights once every 2 weeks, once a week, and multiple times per week, flying once every 2 weeks was insufficient in generating predictions using ground data, whereas multiple flights per week showed diminishing returns in facilitating prediction, concluding that a single flight per week had accuracy on par with two or three flights per week. This likely differs by crop, environment, and measured trait. Optimization of data acquisition, processing, and presenting will lower barriers to entry and grant more equal access to phenomics-guided precision agricultural practices.

## Conclusion

This study revealed that (i) temporal data derived from multiple drone surveys could predict southern rust and senescence variation occurring within late reproductive stages in maize, (ii) machine learning regressions outperformed the simple linear model in predicting unknown genotypes in target environments using temporal phenomic data, (iii) nested design revealed that temporal traits constructing the phenomic data were temporally heritable, and (iv) temporal senescence scored by drone images revealed critical positive correlations between the grain filling period and yield in maize.

## Supplementary Information

Supplementary Information 1.

Dataset S1.

Dataset S2.

Dataset S3.
