# Supplementary material for: Phenomic data-facilitated rust and senescence prediction in maize using machine learning algorithms
Source: Sci Rep. 2022 May 9;12:7571. doi: 10.1038/s41598-022-11591-0 (PMC9085875; doi:10.1038/s41598-022-11591-0)
Supplement: Supplementary file 1 — Supplementary Information 1. [file 41598_2022_11591_MOESM1_ESM.docx]

**Supplementary Information**

**Supplementary Figure 1**


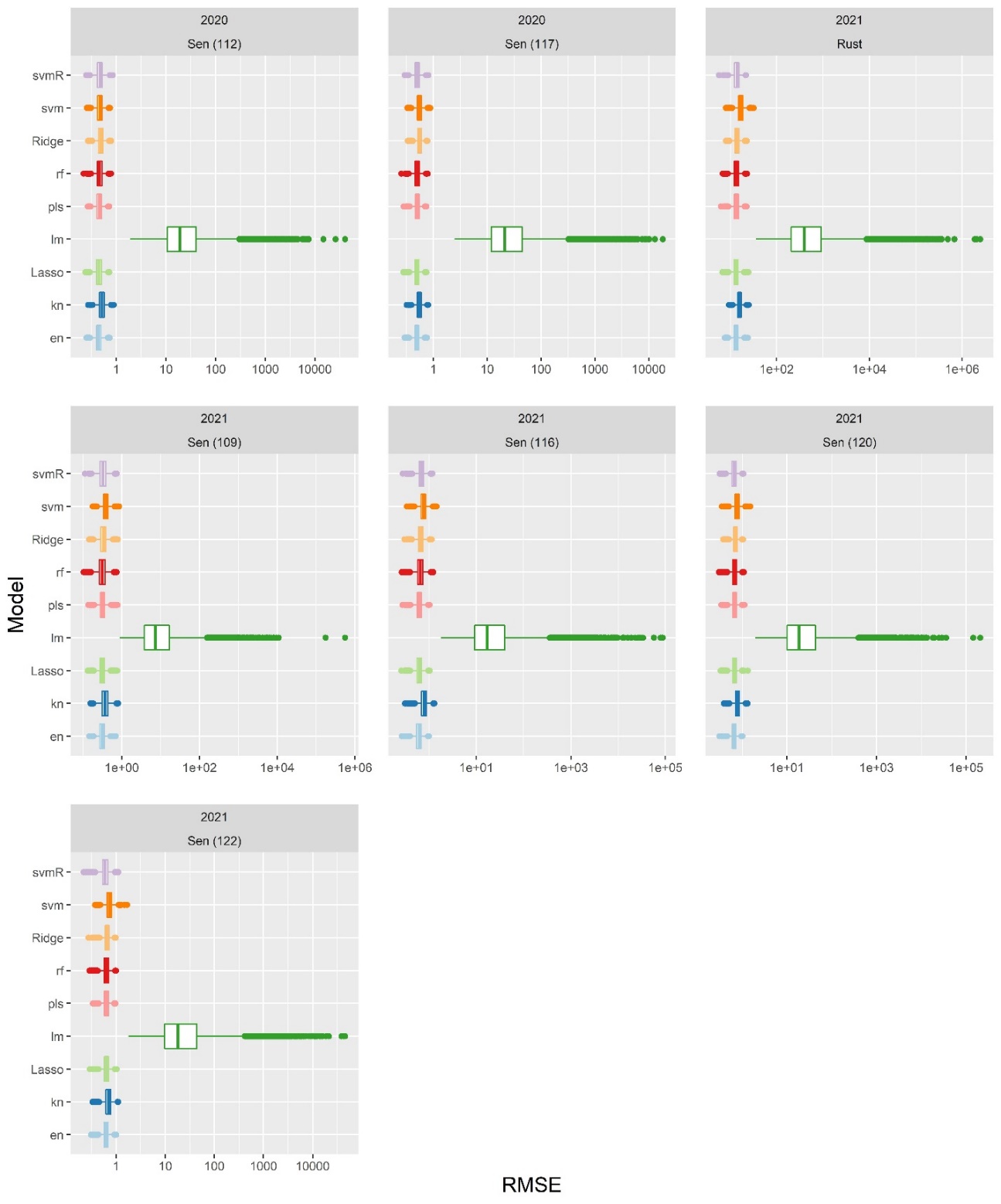


**Supplementary Figure 1.** Root mean square error (RMSE) values are displayed for each machine learning regression alongside the linear model for each predicted trait. Each model was evaluated 500 times across all seven predicted traits; the spread of RMSE values is represented as a boxplot for each model. Individual plots are named according to the year for which that trait was predicted along with the trait’s name (example: 2020 Sen(112) refers to the 2020 planting season, and specifically senescence at 112 days after planting).

**Supplementary Figure 2**


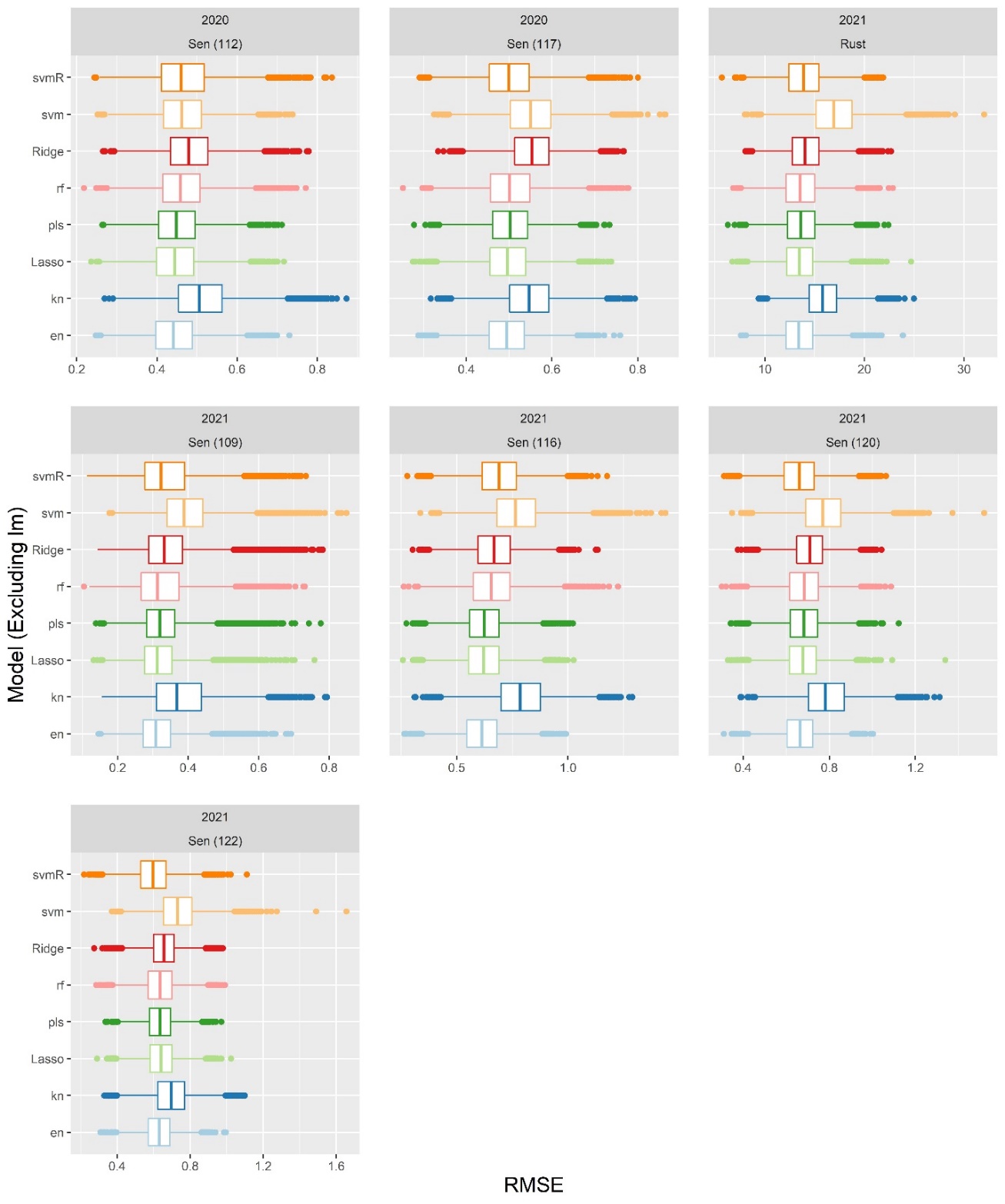


**Supplementary Figure 2**. RMSE spreads are shown strictly for machine learning models (the linear model is excluded for better comparison between machine learning models). Each model was evaluated 500 times across each of seven predicted traits.

**Supplementary Table 1**. Vegetation indices used in this study with their formulae and references.

| **Vegetation Index (VI)** | **Formula** | **Reference(s)** |
| --- | --- | --- |
| Brightness index (BI) | $sqrt(\frac{R^{2}+G^{2}+B^{2}}{3})$ | Richardson et al., 1977^1^ |
| Green leaf index (GLI) | $\frac{2*G-R-B}{2*G+R+B}$ | Louhaichi et al., 2001^2^ |
| Normalized green-red difference index (NGRDI) | $\frac{G-R}{G+R}$ | Tucker, 1979^3^ |
| Visible atmospherically resistant index (VARI) | $\frac{G-R}{G+R-B}$ | Gitelson et al., 2002^4^ |
| Blue green pigment index (BGI) | $\frac{B}{G}$ | Zarco-Tejada et al., 2005^5^ |
| Blue chromatic coordinate index (BCC) | $\frac{B}{R+G+B}$ | Woebbecke et al., 1995^6^ |
| Color index of vegetation extraction (CIVE) | $0.441R-0.811G+$  $0.385B+18.78745$ | Kataoka et al., 2003^7^ |
| Combined indices 1 (COM1) | $EXG+CIVE+EXGR+VEG$ | Guijarro et al., 2011^8^ |
| Combined indices 2 (COM2) | $0.36EXG+0.47CIVE+0.17VEG$ | Guerrero et al., 2012^9^ |
| Excessive green (ExG) | $\left( 2*G \right)-R-B$ | Woebbecke et al., 1995^6^ |
| Normalized excess green index (ExG2) | $\frac{2G-R-B}{G+R+B}$ | Woebbecke et al., 1995^6^ |
| Excess green minus excess red index (ExGR) | $\left( 3*G \right)-\left( 2.4*R \right)-B$ | Meyer & Neto, 2008^10^ |
| Excessive red (EXR) | $1.4R-G$ | Meyer et al., 1999^11^ |
| Green minus blue index (G-B) | $G-B$ | Woebbecke et al., 1995^6^ |
| Green minus red index (G-R) | $G-R$ | Woebbecke et al., 1995^6^ |
| Green blue simple ratio index (G/B) | $\frac{G}{B}$ | Woebbecke et al., 1995^6^ |
| Green red simple ratio index (G/R) | $\frac{G}{R}$ | Woebbecke et al., 1995^6^ |
| Green chromatic coordinate index (GCC) | $\frac{G}{R+G+B}$ | Woebbecke et al., 1995^6^ |
| Modified excess green index (MExG) | $1.262G-0.884R-0.311B$ | Burgos-Artizzu et al., 2011^12^ |
| Modified green red index (MGVRI) | $\frac{G^{2}- R^{2}}{G^{2}+ R^{2}}$ | Bendig et al., 2015^13^ |
| Normalized difference index (NDI) | $128*\left( \left( \frac{G-R}{G+R} \right)+1 \right)$ | Hamuda et al., 2016^14^ |
| Normalized difference red blue index (NDRBI) | $\frac{R-B}{R+B}$ | Golzarian & Frick, 2011^15^ |
| Normalized green-blue difference index (NGBDI) | $\frac{G-B}{G+B}$ | Hunt et al., 2005^16^ |
| Red minus blue index (R-B) | $R-B$ | Woebbecke et al., 1995^6^ |
| Red blue simple ratio index (R/B) | $\frac{R}{B}$ | Woebbecke et al., 1995^6^ |
| Red chromatic coordinate index (RCC) | $\frac{R}{R+G+B}$ | Woebbecke et al., 1995^6^ |
| Modified red chromatic coordinate index (MRCC) | $\frac{R^{3}}{R+G+B}$ | Created in this study |
| Red green blue index (RGBVI) | $\frac{G^{2}-R*B}{G^{2}+R*B}$ | Bendig et al., 2015^13^ |
| Triangular greenery index (TGI) | $G-\left( 0.39R 0 0.69B \right)$ | Hunt et al., 2011^17^ |
| Vegetative (VEG) | $\frac{G}{R^{0.667}*B^{0.334}}$ | Hague et al., 2006^18^ |
| Normalized red minus blue index (NRMBI) | $\frac{R-B}{G}$ | Created in this study |
| Modified Simple Ratio Green and Red (MSRGR) | $sqrt\left( \frac{G}{R} \right)$ | Tucker, 1979^3^ |
| Transformed normalized green and red (TNDGR) | $sqrt\left( \left( \frac{G-R}{G+R} \right)+0.5 \right)$ | Tucker, 1979^3^ |

**Supplementary Table 2**. Connecting letters reports from student’s *t*-tests of phenomic predictive model accuracies.

| CV2 DTA 2020 | Mean | Letter |
| --- | --- | --- |
| rf | 0.68 | A |
| svmR | 0.68 | B |
| lasso | 0.67 | B |
| en | 0.67 | B |
| pls | 0.66 | C |
| ridge | 0.65 | D |
| svm | 0.64 | E |
| knn | 0.61 | F |
| lm | 0.02 | G |
|  |  |  |
| CV2 DTS 2020 | Mean |  |
| rf | 0.68 | A |
| svmR | 0.68 | A |
| lasso | 0.67 | B |
| en | 0.67 | B |
| pls | 0.65 | C |
| ridge | 0.65 | D |
| svm | 0.64 | D |
| knn | 0.61 | E |
| lm | 0.02 | F |
|  |  |  |
| CV2 EHT 2020 | Mean |  |
| en | 0.50 | A |
| ridge | 0.49 | AB |
| pls | 0.49 | B |
| lasso | 0.47 | C |
| rf | 0.46 | C |
| svm | 0.42 | D |
| svmR | 0.41 | E |
| knn | 0.33 | F |
| lm | 0.00 | G |
|  |  |  |
| CV2 FHT 2020 | Mean |  |
| en | 0.76 | A |
| lasso | 0.76 | A |
| pls | 0.76 | A |
| ridge | 0.75 | B |
| rf | 0.74 | C |
| svm | 0.73 | D |
| svmR | 0.72 | E |
| knn | 0.70 | F |
| lm | 0.02 | G |
|  |  |  |
| CV2 PHT 2020 | Mean |  |
| en | 0.77 | A |
| lasso | 0.77 | A |
| pls | 0.77 | A |
| ridge | 0.76 | B |
| rf | 0.75 | B |
| svm | 0.73 | C |
| svmR | 0.73 | C |
| knn | 0.70 | D |
| lm | 0.03 | E |
|  |  |  |
| CV2 Senescence_07072020 2020 | Mean |  |
| en | 0.70 | A |
| lasso | 0.70 | A |
| svm | 0.69 | B |
| pls | 0.67 | C |
| rf | 0.67 | D |
| svmR | 0.65 | E |
| ridge | 0.65 | E |
| knn | 0.55 | F |
| lm | 0.02 | G |
|  |  |  |
| CV2 Senescence_07122020 2020 | Mean |  |
| lasso | 0.77 | A |
| en | 0.77 | A |
| rf | 0.77 | B |
| svmR | 0.77 | B |
| pls | 0.76 | C |
| svm | 0.73 | D |
| ridge | 0.72 | E |
| knn | 0.71 | F |
| lm | 0.03 | G |
|  |  |  |
| CV2 YLD2 2020 | Mean |  |
| en | 0.64 | A |
| ridge | 0.64 | A |
| svm | 0.64 | A |
| svmR | 0.64 | AB |
| rf | 0.64 | AB |
| knn | 0.63 | ABC |
| pls | 0.63 | BC |
| lasso | 0.62 | C |
| lm | 0.02 | D |
|  |  |  |
| CV2 DTA 2021 | Mean |  |
| ridge | 0.65 | A |
| en | 0.64 | AB |
| lasso | 0.64 | BC |
| rf | 0.63 | CD |
| pls | 0.63 | DE |
| svmR | 0.62 | DE |
| svm | 0.62 | E |
| knn | 0.61 | F |
| lm | 0.07 | G |
|  |  |  |
| CV2 DTS 2021 | Mean |  |
| ridge | 0.72 | A |
| en | 0.72 | A |
| rf | 0.71 | B |
| pls | 0.71 | B |
| knn | 0.71 | B |
| svmR | 0.70 | BC |
| lasso | 0.70 | C |
| svm | 0.66 | D |
| lm | 0.07 | E |
|  |  |  |
| CV2 EHT 2021 | Mean |  |
| en | 0.55 | A |
| ridge | 0.54 | A |
| lasso | 0.52 | B |
| pls | 0.52 | BC |
| rf | 0.51 | C |
| svmR | 0.49 | D |
| knn | 0.44 | E |
| svm | 0.40 | F |
| lm | 0.02 | G |
|  |  |  |
| CV2 FHT 2021 | Mean |  |
| en | 0.77 | A |
| lasso | 0.77 | AB |
| pls | 0.76 | BC |
| ridge | 0.76 | CD |
| rf | 0.75 | D |
| svmR | 0.74 | E |
| svm | 0.69 | F |
| knn | 0.69 | F |
| lm | 0.04 | G |
|  |  |  |
| CV2 PHT 2021 | Mean |  |
| en | 0.76 | A |
| lasso | 0.76 | A |
| pls | 0.75 | B |
| ridge | 0.74 | BC |
| rf | 0.74 | C |
| svmR | 0.72 | D |
| svm | 0.71 | E |
| knn | 0.67 | F |
| lm | 0.03 | G |
|  |  |  |
| CV2 Rust 2021 | Mean |  |
| rf | 0.72 | A |
| en | 0.7 | B |
| lasso | 0.7 | B |
| ridge | 0.69 | C |
| svmR | 0.69 | C |
| pls | 0.68 | C |
| svm | 0.62 | D |
| knn | 0.59 | E |
| lm | 0.04 | F |
|  |  |  |
| CV2 Senescence_71621 | Mean |  |
| lasso | 0.73 | A |
| en | 0.73 | A |
| rf | 0.71 | B |
| pls | 0.69 | C |
| svmR | 0.67 | D |
| ridge | 0.67 | D |
| svm | 0.64 | E |
| knn | 0.61 | F |
| lm | 0.03 | G |
|  |  |  |
| CV2 Senescence_72321 | Mean |  |
| lasso | 0.79 | A |
| en | 0.79 | A |
| pls | 0.77 | B |
| rf | 0.76 | C |
| ridge | 0.76 | C |
| svmR | 0.73 | D |
| svm | 0.71 | E |
| knn | 0.66 | F |
| lm | 0.03 | G |
|  |  |  |
| CV2 Senescence_72721 | Mean |  |
| svmR | 0.8 | A |
| en | 0.8 | A |
| lasso | 0.8 | A |
| rf | 0.79 | A |
| ridge | 0.78 | B |
| pls | 0.78 | B |
| svm | 0.77 | C |
| knn | 0.71 | D |
| lm | 0.06 | E |
|  |  |  |
| CV2 Senescence_72921 | Mean |  |
| svmR | 0.75 | A |
| rf | 0.73 | B |
| lasso | 0.73 | B |
| en | 0.73 | B |
| pls | 0.73 | B |
| ridge | 0.71 | C |
| svm | 0.7 | D |
| knn | 0.66 | E |

**Supplementary Table 3**. Comparisons between traditional and UAS-based phenotyping methods.

|  | **Traditional field-based phenotyping** | **UAS-based high throughput phenotyping** |
| --- | --- | --- |
| **Cost** | Hourly wages for personnel. Specialized personnel are not necessary but likely improve accuracy. | Phantom 4 Pro v2 Drone: $1500  Agisoft Metashape Professional (Education Edition): $549/year  GPS for ground control points: $1000+  Desktop computer with adequate CPU/GPU/RAM/storage: $2000+ |
| **Time** | Scoring and recording data for 200 hybrids requires between 2-2.5 hours of time for one rater (provided field is dry enough for walking). | Each UAS flight takes roughly 30 minutes; mosaicking process requires between 4 to 8 hours to stitch images together (mostly computational, approximately 2 hours of manual input); and manual scoring of each hybrid from a mosaic requires approximately 6 hours per mosaic. |
| **Accuracy** | Rater bias is subject to level of expertise, environmental factors, lighting conditions, weather, and fatigue. | Orthomosaics provide uniform arrangement of plants to be scored; subject to lighting conditions during UAS flight, quality of image stitching, and (to a lesser degree) rater bias. |
| **Resolution** | No image resolution limitations, however limited to lower and mid-level leaves at the end of plot, the interior of plots is more challenging to observe; canopy can be difficult to phenotype when plants are at maximum height. | Pixel size of mosaics is determined by UAS flying height, speed and camera resolution and ability; visible plant area restricted to canopy and some mid-level leaves. |
| **Scale** | Limited by available and trained personnel in the field. | Limited by the available number of batteries for UAS and willingness of operator in the field. Limited by available and trained personnel in the lab. |

**References**

1 Richardson, A. J. & Wiegand, C. L. Distinguishing vegetation from soil background information. *Photogrammetric engineering and remote sensing* **43**, 1541-1552 (1977).

2 Louhaichi, M., Borman, M. M. & Johnson, D. E. Spatially located platform and aerial photography for documentation of grazing impacts on wheat. *Geocarto International* **16**, 65-70 (2001).

3 Tucker, C. J. Red and photographic infrared linear combinations for monitoring vegetation. *Remote sensing of Environment* **8**, 127-150 (1979).

4 Gitelson, A. A., Kaufman, Y. J., Stark, R. & Rundquist, D. Novel algorithms for remote estimation of vegetation fraction. *Remote sensing of Environment* **80**, 76-87 (2002).

5 Zarco-Tejada, P. J. *et al.* Assessing vineyard condition with hyperspectral indices: Leaf and canopy reflectance simulation in a row-structured discontinuous canopy. *Remote Sensing of Environment* **99**, 271-287 (2005).

6 Woebbecke, D. M., Meyer, G. E., Von Bargen, K. & Mortensen, D. A. Color indices for weed identification under various soil, residue, and lighting conditions. *Transactions of the ASAE* **38**, 259-269 (1995).

7 Kataoka, T., Kaneko, T., Okamoto, H. & Hata, S. b1079-b1083 (IEEE).

8 Guijarro, M. *et al.* Automatic segmentation of relevant textures in agricultural images. *Computers and Electronics in Agriculture* **75**, 75-83 (2011).

9 Guerrero, J. M., Pajares, G., Montalvo, M., Romeo, J. & Guijarro, M. Support vector machines for crop/weeds identification in maize fields. *Expert Systems with Applications* **39**, 11149-11155 (2012).

10 Meyer, G. E. & Neto, J. C. Verification of color vegetation indices for automated crop imaging applications. *Computers and electronics in agriculture* **63**, 282-293 (2008).

11 Meyer, G. E., Hindman, T. W. & Laksmi, K. 327-335 (International Society for Optics and Photonics).

12 Burgos-Artizzu, X. P., Ribeiro, A., Guijarro, M. & Pajares, G. Real-time image processing for crop/weed discrimination in maize fields. *Computers and Electronics in Agriculture* **75**, 337-346 (2011).

13 Bendig, J. *et al.* Combining UAV-based plant height from crop surface models, visible, and near infrared vegetation indices for biomass monitoring in barley. *International Journal of Applied Earth Observation and Geoinformation* **39**, 79-87 (2015).

14 Hamuda, E., Glavin, M. & Jones, E. A survey of image processing techniques for plant extraction and segmentation in the field. *Computers and Electronics in Agriculture* **125**, 184-199 (2016).

15 Golzarian, M. R. & Frick, R. A. Classification of images of wheat, ryegrass and brome grass species at early growth stages using principal component analysis. *Plant Methods* **7**, 1-11 (2011).

16 Hunt, E. R., Cavigelli, M., Daughtry, C. S. T., McMurtrey, J. E. & Walthall, C. L. Evaluation of digital photography from model aircraft for remote sensing of crop biomass and nitrogen status. *Precision Agriculture* **6**, 359-378 (2005).

17 Hunt, E. R., Daughtry, C. S. T., Eitel, J. U. H. & Long, D. S. Remote sensing leaf chlorophyll content using a visible band index. (2011).

18 Hague, T., Tillett, N. D. & Wheeler, H. Automated crop and weed monitoring in widely spaced cereals. *Precision Agriculture* **7**, 21-32 (2006).
